# Dispensing of psychotropic drugs in the Brazilian capital city before and during the COVID-19 pandemic (2018–2020)

**DOI:** 10.3389/fphar.2022.1028233

**Published:** 2022-12-23

**Authors:** Pamela Alejandra Escalante Saavedra, Dayani Galato, Calliandra Maria de Souza Silva, Izabel Cristina Rodrigues da Silva, Emília Vitoria da Silva

**Affiliations:** ^1^ Federal Pharmaceutical Council, Brasília, Brazil; ^2^ Faculty of Ceilândia, University of Brasília, Brasília, Brazil; ^3^ Collaborating researcher of the University of Brasília, Brasília, Brazil

**Keywords:** psychotropic drugs, products commerce, community pharmacy services, COVID-19, mental health assistance

## Abstract

**Objective:** Evaluate the data on the psychotropic drugs dispensed by private community pharmacies before and during the SARS-CoV-2 pandemic.

**Methods:** This cross-sectional study compared the quarterly and annual consumption of psychotropic drugs per Defined Daily Dose per 1000 inhabitants-day (DHD). Interrupted time series were also constructed to expose changes in the consumption pattern in the periods before and after March 2020.

**Results:** Among the 20 most consumed psychoactive drugs, 12 were antidepressants, for example, escitalopram (DHD 7.996 and 10.626; *p* < 0.001), and sertraline (DHD 6.321 and 8.484; *p* < 0.001), in addition to the hypnotic zolpidem (DHD 6.202 and 8.526; *p* < 0.001). The time series reveals (*R* ^2^ value) a variation in drug dispensing, in DHD values, during the pandemic.

**Conclusion:** Despite the higher variance, a significant increase is clearly seen in the consumption trends of psychoactive drugs, particularly antidepressants, consistent with the pandemic’s influence on the general population’s mental health.

## 1 Introduction

Psychotropic drug use has shown a growing trend in recent decades in several countries, including Brazil (Rodrigues et al., 2020). At the country level, the trade of these drugs represents a significant part of consumption (Oliveira et al., 2021). Their safety profile presents the potential to cause several adverse events; for example, the withdrawal symptoms associated with benzodiazepines, Z-drugs, ketamine, selective serotonin reuptake inhibitors (SSRIs), serotonin-norepinephrine reuptake inhibitors (SNRIs), tricyclic antidepressants (TCAs), antipsychotics, monoamine oxidase inhibitors (MAOIs), and gabapentin (Cosci and Chouinard, 2020). Therefore, their sales and dispensing is essential and, consequently, is regulated by the Ministry of Health and the Brazilian Health Regulatory Agency (Anvisa) (Brasil, 1998; Brazilian Ministry of Health, Brazil, 2020b).

Anvisa developed the National System for the Management of Controlled Products (SNGPC) database in 2007 to initially monitor the dispensing of psychotropic medications and narcotic substances and their precursors. In 2010, the SNGPC included antimicrobials in the monitoring, and this control allowed the monitoring of prescription habits and controlled substance consumption (Brazilian Ministry of Health, Brazil, 2020b). In November 2020, the Agency allowed the public access to the SNGPC to consult and extract all the information available in this database (Brazilian Ministry of Health, Brazil, 2020a), which has made it possible to analyze and evaluate trends in the sales and the dispensing of various medicines at the local and national level.

Epidemiological studies can generate quality and reliable information on how and to what extent the pandemic has influenced the mental state of communities. Although the utilization of antidepressants, anxiolytics, and hypnotics was high in Brazil, mainly to benzodiazepines and Z-drugs (Madruga et al., 2019; Curado et al., 2022), COVID-19’s pandemic affects the individuals' physical health, the pandemic’s social restriction measures have impacted people’s mental health (COVID-19 Mental Disorders Collaborators, 2021; Organização Pan American Health Organization, 2020; Saavedra et al., 2020).

For instance, the COVID-19 Mental Disorders Collaborators' systematic review analyzed 204 countries and territories in 2020 and revealed, among others, that people’s reduced mobility was associated with an increase in major depressive disorder (*p* = 0.029) and anxiety (*p* = 0.0001) prevalence (COVID-19 Mental Disorders Collaborators, 2021). Among the psychotropic drugs regulated in Brazil that could highlight the potential and actual consequences of the COVID-19 pandemic on people’s mental health are antidepressants and anxiolytics (Madruga et al., 2019; Curado et al., 2022).

Of the numerous ways to understand the COVID-19 pandemic’s impact on mental health, measuring psychotropic drugs' dispensing could become a timely public health tool. Hence, this study evaluated these prescriptions and dispensing by private community pharmacies data before and during the SARS-CoV-2 pandemic in the Brazilian Capital, Federal District.

## 2 Methods

### 2.1 Study database

We performed a cross-sectional study with retrospective data collection through the Brazilian Open Data Portal (Brazilian Ministry of Health, Brazil, 2020a) based on secondary data from the National System for the Management of Controlled Products (SNGPC), available by the Brazilian Health Regulatory Agency (Anvisa) and is updated monthly (Brasil. 2020a). As this research was conducted in open databases, approval by an Ethics Research Committee is not required in Brazil (National Health Council, Brazil, 2012).

National data corresponding to the period from 1 January 2018, to 30 December 2020, were collected, and a subsample was built corresponding to Brasília (the Brazilian Capital City located in the Federal District). From this subsample, we extracted the variables related to the registration of commercialization (month and year of the sale); related to the drug (active ingredient, pharmaceutical form, concentration per dosage unit in mg); and related to dispensing (number of dosage units per secondary packaging and quantity of secondary packaging dispensed). Incomplete or inconsistent records were discarded (data cleansing). The evaluated drugs received an Anatomical Therapeutic Chemical (ATC) Classification code, referring to group N–Nervous System.

### 2.2 Database analysis

We determined the Defined Daily Dose per 1000 inhabitants-day (DHD)^9^ for each evaluated drug, the DHD adjusted (Capellá and Laporte, 1993) by month and quarter for the years chosen by this study (2018–2020), and the defined daily dose (DDD) established by the World Health Organization WHOCC - ATC/DDD Index (available at: https://www.whocc.no/atc_ddd_index/). Although the dispensing record does not necessarily indicate the actual consumption of psychotropic drugs by the population, for this study, we considered that the dispensing represents an estimation of the consumption data of the study participants. This dispensing-consumption date equivalence is because the study design does not allow us to confirm that the patient actually used the dispensed medication. The included drugs' DHDs were compared monthly, quarterly, and yearly to help identify seasonality patterns before and during the pandemic.

### 2.3 Statistical analysis

This study evaluated consumption trends (DHD of each drug) compared to data from the literature, with March 2020 marking the health phenomenon threshold due to it being the temporary alteration of regular day-a-day life start and the first COVID-19 cases recorded in the Federal District. The DHD data were expressed in absolute values and percentage changes (percentage change in the median DHD of the period after February 2020 compared to the median DHD before February 2020). The Mann-Whitney U test was used to calculate the *p*-value, considering values of *p* < 0.05 to be significant.

Likewise, interrupted time series were constructed to expose changes in the consumption pattern of the drugs under study. The linear regression and their R2 values, with a 95% confidence interval, were calculated for each evaluated drug.

R, SPSS (version 28.0.1), and Microsoft Excel^®^ software were used for the data analysis.

## 3 Results

Of the total records found between 1 January 2018, and 30 December 2020, the dispensing of controlled drugs that occurred in the Brazilian Capital’s private community pharmacies corresponded to 1.3% of the national total (n = 2,798,195), with the majority (81%) being anti-infectives for systemic use and 19% of medications were for the central nervous system (532,074 records).

**FIGURE 1:**
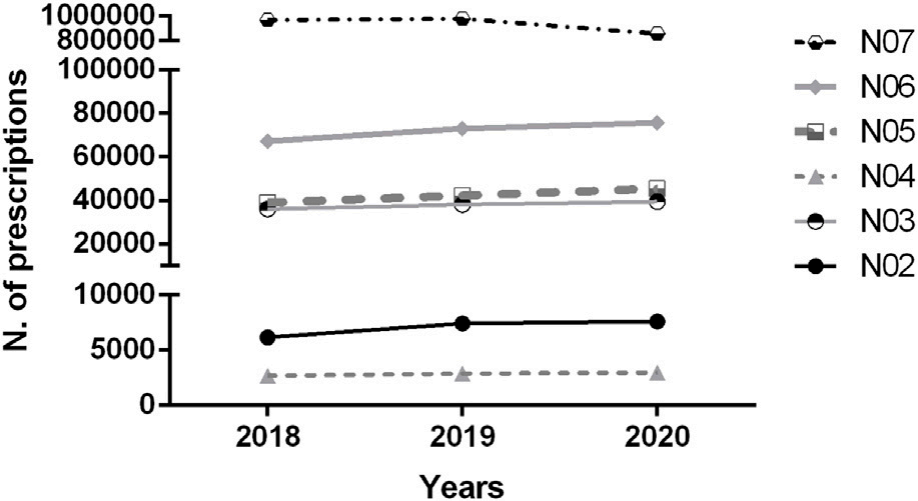
The dispensing records’ total number in the National System for the Management of Controlled Products (SNGPC) database distributed by therapeutic subgroup (nervous system) in the Federal District Brazil 2018–2020. N02: Analgesics; N03: Antiepileptics; N04: Anti-Parkinson drugs; N05: Psycholeptics; N06: Psychoanaleptics; N07: Other drugs that act on the Nervous System.

Figure 1 shows the total number of prescriptions according to the Anatomical Therapeutic Chemical (ATC) classification’s therapeutic subgroup of N (Nervous System) plain drugs corresponding to dispensations made in the years 2018, 2019, and 2020. Drugs from subgroup N01 (anesthetics; 0.1% of the total) do not appear in this graph.

Table 1 presents the total consumption, by year, of the 20 medications with the highest Defined Daily Dose per 1000 inhabitants-day (DHD) in the Federal District, according to 2019 consumption. The data corresponding to 2018 and 2020 is also presented, allowing comparisons between the periods analyzed (in terms of DHD).

**TABLE 1:** List and evolution of the 20 psychotropic medications with the highest DHD, base year 2019, registered in the National System for the Management of Controlled Products (SNGPC) database in the Federal District, Brazil, 2018–2020.

ATC	Active ingredient	DDD (mg)	DHD 2018	DHD 2019	DHD 2020	*p*-value
N06AB10	ESCITALOPRAM	10	7.996	9.483	10.626	<0.001*
N06AB06	SERTRALINE	50	6.321	7.715	8.484	<0.001*
N05CF02	ZOLPIDEM	10	6.202	7.507	8.526	<0.001*
N06AB03	FLUOXETINE	20	6.583	5.269	5.163	0.021*
N06AX23	DESVENLAFAXINE	50	3.466	4.662	5.4	<0.001*
N05BA12	ALPRAZOLAM	1	3.217	3.352	3.305	0.178
N06AX21	DULOXETINE	60	2.653	3.017	3.16	<0.001*
N06AX16	VENLAFAXINE	100	2.281	2.645	2.932	<0.001*
N06AB05	PAROXETINE	20	2.120	2.296	2.375	0.001
N06AX12	BUPROPIONE	300	1.949	2.206	2.245	0.002*
N06AB04	CITALOPRAM	20	1.589	1.791	1.68	0.569
N03AA02	PHENOBARBITAL	100	2.159	1.714	1.24	<0.001*
N05AX08	RISPERIDONE	5	1.221	1.398	1.273	0.204
N06BA04	METHYLPHENIDATE	30	1.247	1.355	1.089	0.006*
N06AX11	MIRTAZAPINE	30	1.185	1.35	1.462	<0.001*
N03AE01	CLONAZEPAM	8	1.261	1.269	1.191	0.043*
N06BA12	LISDEXAMPHETAMINE	30	0.910	1.254	1.322	<0.001*
N06AA09	AMITRIPTYLINE	75	1.368	1.063	0.902	0.066
N06AX05	TRAZODONE	300	0.877	1.021	1.168	<0.001*
N03AX16	PREGABALIN	300	0.801	0.985	1.136	<0.001*

DDD, defined daily dose; DHD, Defined Daily Dose per 1000 inhabitants-day. * Mann-Whitney U test with a *p* < 0.05.

We highlight that among the 20 medications with the highest DHD according to consumption in 2019, 12 are antidepressants, of which ten showed a trend towards a statistically significant increase in consumption when comparing the groups analyzed - median DHD before and after March 2020 (Table 1).

The escitalopram DHD was between 7.99 and 10.62, which means that more than nine doses were dispensed each day for treatment and, similarly, the sertraline DHD variation was between 6.32 and 8.48, with more than seven doses dispensed per day in the same period (Table 1).

Zolpidem (sedative-hypnotic drug) stands out as its DHD increased from 6.20 to 8.52 (2018 and 2020), with a percentage change of 22.6% in consumption when comparing the periods before and after March 2020 (Table 1; *p* < 0.001).

Among the medications, negative percentage changes occurred for fluoxetine (−3.8%), clonazepam (−7.2%), amitriptyline (−16.5%), phenobarbital (−41.0%), and methylphenidate (−19.5%), i.e., a lower median DHD in 2020 related to the median DHD in 2018. Except for amitriptyline, this decrease was statistically significant.

**FIGURE 2:**
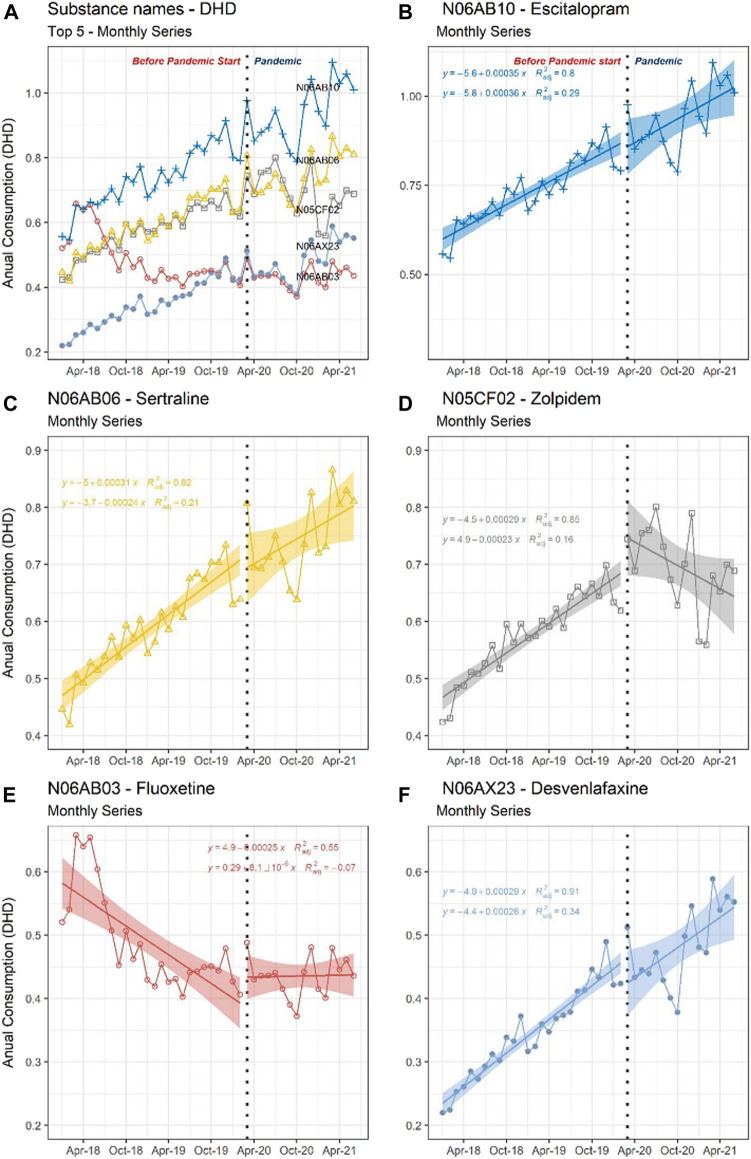
Interrupted time series of the five most dispended medications, 2018 to 2020, with sanitary interruption marked in March 2020. The vertical axis displays the monthly consumption measured in adjusted DHD, and the horizontal axis displays the time measured in calendar months. The consumption trend of each drug is presented based on linear regression (95% CI) and the R2 value. The top 5 medication consumption trends are shown in graph **(A)**; these Top 5 separated consumption trends are shown in graphs **(B–F)**: N06AB10 - Escitalopram **(B)**, N06AB06 - Sertraline **(C)**, N05CF02 - Zolpidem **(D)**, N06AB03 - Fluoxetine **(E)**, and N06AX23 - Desvenlafaxine. DHD, Defined Daily Dose per 1000 inhabitants-day.

Figure 2 displays the consumption evolution of the five drugs with the highest DHD (escitalopram, sertraline, zolpidem, fluoxetine, and desvenlafaxine) before and during the pandemic. We consider the five high doses dispensed, measured by DHD, for selecting the drugs. The temporary sanitary interruption was marked as March 2020 (see the Methodology section). These interrupted time series present the monthly accumulated adjusted DHD. Figures 2B–F display the same series as Figure 2 individually to highlight *R* ^2^ and confidence interval (shaded CI) data pertinent to each drug.

Figure 2 identifies, on the vertical (Y) axis, the monthly consumption measured in adjusted DHD and, on the horizontal (X) axis, the time measured in calendar months; therefore, the accumulated DHD in the month is measured. Figures 2B–F display the same series as Figure 2 individually to highlight *R* ^2^ and confidence interval (shaded CI) data pertinent to each drug.

## 4 Discussion

The opening of Anvisa’s SNGPC database to the public in Brazil allows for accurate data in the context of primary care crucial for establishing strategies to promote the rational use of medicines in the country, especially during the COVID-19 pandemic. After evaluating the data on psychotropic drugs' prescription and dispensing by private community pharmacies before and during the pandemic (2018–2020), our results showed that the most frequent therapeutic subgroups were psychoanaleptics and psycholeptics, with antidepressants and anxiolytics standing out, with their increased use after March 2020.

Data from the literature on Brazilians' mental health corroborate our prescription pattern as depression incidence has risen since 2003, with self-reported depression rising from 3.9% to 4.1% in 2008, reaching 6.1% in 2014 (Brazil, 2016). Similarly, the Brazilian Ministry of Health released the first results from their research on mental health during the COVID-19 pandemic, revealing that anxiety was the most present disorder in the period, followed by post-traumatic stress disorder and a low proportion of depression in its most severe form (Brazilian Ministry of Health, Brazil, 2020c).

This trend was possibly affected by the pandemic phenomenon declared in Brazil in March 2020. The mobility restrictions, social distancing, and quarantine imposed on people by the health authorities to reduce the spread of the virus most likely impacted the mental health and wellbeing of the population.

Furthermore, in a general Brazilian population study, Goularte et al. (2021) investigated the emergence and determinants of psychiatric symptoms during the pandemic through an online interview. They found that the most frequently described conditions the interviewees reported were anxiety (81.9%), depression (68.0%), somatic symptoms (62.6%), and sleep problems (55.3%). The authors concluded that, given the high prevalence of psychiatric symptoms observed, the impact of the COVID-19 pandemic on people’s mental health should be considered a public health problem in Brazil (Goularte et al., 2021).

Similarly, a cross-sectional quantitative study conducted by Passos et al. (2020) and based on an online questionnaire, which was constructed with four domains, one specific to generalized anxiety disorders (Generalized Anxiety Disorder-7), to evaluate the psychiatric symptoms of Brazilian and Portuguese individuals. Their results revealed a prevalence of 71.3% for anxiety, 24.7% for depression, and 23.8% for individuals with both depression and anxiety, concluding that mental illness was considerably higher than prior to the COVID-19 pandemic (Passos et al., 2020). These data suggest that sanitary deterioration could aggravate Brazilians' psychotropics use.

Before the COVID-19 pandemic, the increased use of psychopharmaceuticals had already been identified in Brazil. Oliveira et al. (2021)’s study in Ribeirão Preto, a city of 720 thousand inhabitants, described the Primary Care patients' consumption of psychotropic drugs and found that sertraline, clonazepam, and risperidone presented a higher consumption growth than the city’s population growth rate between 2008 and 2012 (Oliveira et al. (2021).

These results collaborate with Hoefler et al. (2022)’s exploratory ecological study that found antidepressant sales in Brazil from November 2014 to October 2019 increased from 23, 3 DHD in 2014 to 38, 3 DHD in 2019 (*p* = 0.002) and that the selective serotonin reuptake inhibitors were the most sold antidepressant category (Hoefler et al., 2022).

Psychotropic medications are frequently prescribed in Brazil´s Federal District. The SNGPC data analyzed from 2018 to 2020 revealed an expressive increase in antidepressants after March 2020, particularly for desvenlafaxine (38.9%), escitalopram (29.1%), and sertraline (24.3%), like the results found by Hoefler et al. (2022) (Hoefler et al., 2022), as well as the hypnosedative (anxiolytic) zolpidem (22.4%) - all statistically significant. These results imply that the isolation and social restriction negatively affected the mental health of the Brazilian Capital’s residents.

Rabeea et al. (2021) analyzed England’s national trends in the prescriptions and costs of several antidepressants during the COVID-19 pandemic. They found a 5% increase in the number of antidepressant dispensations from January to December 2020 with regard to 2019. The sertraline costs corresponded to 81.9% of the English National Health Service (NHS)’s total additional resources during 2020 (£139 million in total, of which 113 million corresponded to sertraline) (Rabeea et al., 2021).

The increase in dispensation and, consequently, in antidepressant consumption presented in our study reaffirms the findings of a Spanish study that sought to identify post-traumatic stress, anxiety, burnout, depression, and resilience levels in health personnel during the COVID-19 pandemic. The results showed that 56.6% of health workers have symptoms of post-traumatic stress disorder, 58.6% of anxiety disorder, 46% of depressive disorder, and 41.1% feel emotionally exhausted (Luceño-Moreno et al., 2020).

Our results show no significant results about benzodiazepine drugs. Nevertheless, when considering clonazepam dosage as an anxiolytic (doses of 1–2 mg) and the Prescribed Daily Dose (DDP) of 1 mg, clonazepam is the most prescribed anxiolytic in Brazil and was the most dispensed drug in 2019, with a 9.87 DHD.

To reinforce this point, Zorzanelli et al. (2019)’s study in Rio de Janeiro confirmed the increase in clonazepam consumption, as both an anticonvulsant and hypnosedative, from 0.35 to 1.97 DHD between 2009 and 2013. This increase is even higher when considering only users over 18 years old using 1 mg - the DHD values rose to 21 (Zorzanelli et al., 2019). Our results show that this increase in consumption trend also continues before and after the COVID-19 pandemic.

The difference found between defined daily dose (DDD) for anticonvulsant use (the main indication in European countries) and DDP for use in anxiety and sleep disorders (indications approved in Brazil) can be partially explained by the commercial availability of pharmaceutical specialties' tablets of 0.5 mg and 2.5 mg of the active ingredient, which suggests therapeutic use and dosage used for the indications authorized in Brazil (Brazilian Ministry of Health, Brazil, 2020b).

Another aspect to consider about clonazepam consumption is the region’s human development index (HDI). In a 2013 study in Rio de Janeiro (Brazil), the authors observed that the highest consumption of this drug occurred in the cities with the highest HDI (3.38 and 4.52 DHD) (Brazilian Ministry of Health, Brazil, 2021a). These results may explain the high consumption found in the Federal District, a region classified with a very high HDI, equal 0.824 (Brazilian Institute of Geography and Statistics. Brazil, 2021b) and possibly more significant access to private assistance.

Among the psycholeptic medications present in smaller numbers in our study, only zolpidem (hypnosedative) presented a 22.4% increase in consumption compared to before March 2020 (DHD 6.202 and 8.526 in 2018 and 2020, respectively).

In the same way, in the adult Canadian population, trends in Z-drug and benzodiazepine use were assessed over 15 years. Their results showed that Z-drugs use increased statistically during the study period - an increase in consumption from 8.2 to 28.6 DHD and prevalence from 2.0% to 4.8%. (Brandt et al., 2019). In Israel, Berman et al. (2017)’s study also identified Z-drug and benzodiazepine use trends between 2005 and 2013. Their results showed a 160% global increase in Z-drug and benzodiazepine in 2013 compared to 2005 (from 10.22 to 22.49 DHD), with zolpidem use increasing to 94% and brotizolam to 83% (Berman et al. (2017).

Zolpidem was introduced in Brazil as a hypnosedative with a better safety profile than benzodiazepines (Barros et al., 2018). Our data also point to an increase in zolpidem use, indicating a possible substitution of the therapy used to treat anxiety, possibly due to advertisements favoring Z-drug prescription.

Since the World Health Organization (WHO) declared a public health emergency in March 2020, the stress levels in the population have increased. Antiporta and Bruni (2020)’s study identified the challenges for mental health in South America in the COVID-19 pandemic context. Among the main barriers reported was access to mental health services, including treatments and other types of care, with the national lockdown measures affecting the majority of health centers at the primary level to stop the spread of the disease (Antiporta and Bruni, 2020).

The COVID-19 Mental Disorders Collaborators' systematic review quantified COVID-19 impact on the prevalence of major depression and anxiety disorders globally and found that these disorders increased during 2020 due to the COVID-19 pandemic (COVID-19 Mental Disorders Collaborators, 2021). Even before the pandemic, these diseases appeared as the leading cause of disease burden worldwide. This pandemic has created an increasing urgency to strengthen mental health systems in most countries (Vindegaard and Benros, 2020; Deng et al., 2021; Wu et al., 2021).

Our findings showed reduced prescription of some psychotropic drugs, such as phenobarbital and methylphenidate. This reduction may be justified by the closure measures and the consequent decrease in access to medical care for other diseases unrelated to COVID-19, mainly chronic diseases. In agreement with this, Chisini et al. (2021)’s ecological study detected a reduction in medical consultations in Brazil, with a sharper drop in May 2020 (Chisini et al., 20,212).^28^ This phenomenon is worrying since it can leave chronic patients without treatment.

Selective serotonin reuptake inhibitors (escitalopram, sertraline, and fluoxetine) represent most drugs dispenseding in private pharmacies in Federal District before and during the COVID-19 pandemic, probably because this category is indicated for treating anxiety (Williams et al., 2017). Our results are expected considering the population’s mental status. Furthermore, it agrees with the results presented in an ecological study, where this category was the main antidepressant sold for a total of 5 years period (Hoefler et al., 2022).

This study’s limitations include employing retrospective data, which might contain incomplete or missing data and filling errors due to lack of data insertion from public services; sanitary violations, such as sales without prescriptions, notably over the internet; variety in the quality of pharmacy attendant training that may contribute to the inability of some clerks to fill in the SNGPC worksheet, which does not happen at the time of dispensing but after, resulting in some of the SNGPC database’s incomplete registration/lost data. This fact made it impossible to correlate prescription data and the different Federal District localities and, thus, observe the HDI influence on the dispensing pattern. The SNGPC database also does not allow evaluation of the rationality behind the psychotropic drugs prescription since the registry does not contain any codification or international classification of diseases and health-related problems for the disease requiring the prescription.

The R2 values during the pandemic point to high variance, i.e., the observed values tended to be more distant from the mean (the distribution more spread) and, therefore, it might be necessary to explore the time series' seasonality components changes in terms of recurrence of social isolation measures.

An important aspect to consider when interpreting this study’s results is that the population analyzed in the denominator of the DHD calculation also includes users of pediatric age, a group for whom the use of these drugs is limited, both as anxiolytics and as hypnotics. This inclusion can influence the results found, which requires caution in applying these results.

## 5 Conclusion

The pandemic’s impact on the mental health of the Federal District population is reflected in the increased psychotropic medication consumption, especially antidepressants and anxiolytics. Despite the restrictions on urban mobility and access to health services, people consumed more psychoactive drugs. The most prominent were, among others, escitalopram and sertraline, drugs with approved indications for depression and anxiety, and zolpidem for insomnia treatment.

This study presents a portrait of the psychotropic medications dispensing profile in the Brazilian Capital, revealing a significant increase in psychoactive drug consumption, especially antidepressants. This increase is consistent with the COVID-19 pandemic’s influence on the overall mental health of the population.

## Data Availability

Publicly available datasets were analyzed in this study. This data can be found here: https://dados.gov.br/dataset?q=anvisa.
